# Incorporation of the number of positive lymph nodes leads to better prognostic discrimination of node-positive early stage cervical cancer

**DOI:** 10.18632/oncotarget.15220

**Published:** 2017-02-09

**Authors:** Juan Zhou, San-Gang Wu, Jia-Yuan Sun, Xu-Lin Liao, Feng-Yan Li, Huan-Xin Lin, Li-Chao Yang, Zhen-Yu He

**Affiliations:** ^1^ Department of Obstetrics and Gynecology, The First Affiliated Hospital of Xiamen University, Xiamen 361003, People's Republic of China; ^2^ Department of Radiation Oncology, Xiamen Cancer Hospital, The First Affiliated Hospital of Xiamen University, Xiamen 361003, People's Republic of China; ^3^ Sun Yat-sen University Cancer Center, State Key Laboratory of Oncology in South China, Department of Radiation Oncology, Collaborative Innovation Center of Cancer Medicine, Guangzhou 510060, People's Republic of China; ^4^ Eye Institute of Xiamen University, Fujian Provincial Key Laboratory of Ophthalmology and Visual Science, Medical College, Xiamen University, Xiamen 361005, People's Republic of China; ^5^ Faculty of Basic Medicine, Medical College, Xiamen University, Xiamen 361005, People's Republic of China

**Keywords:** cervical cancer, positive lymph nodes, TNM, tumor histology, survival

## Abstract

To determine the prognostic value of the number of positive lymph nodes (LNs) in cervical cancer and further stratify patients with positive LNs into multiple risk groups based on analysis of Surveillance Epidemiology and End Results (SEER) program. Patients with cervical cancer who undergo hysterectomy and had pathologically-confirmed positive LNs after lymphadenectomy were identified using the SEER database (1988-2012). Kaplan–Meier survival methods and Cox proportional hazards regression were performed. We included 2,222 patients with the median number of removed LNs and positive LNs was 22 and 2, respectively. Multivariable Cox analysis showed patients with > 2 positive LNs had poorer cause-specific survival (CSS) (hazard ratio [HR] 1.631, 95% confidence interval [CI] 1.382–1.926, *P* < 0.001) and overall survival (OS) (HR 1.570, 95% CI 1.346–1.832, *P* < 0.001) than patients with 1–2 positive LNs. Five-year CSS and OS were 78.9% vs. 65.5% (*P* < 0.001) and 76.7% vs. 62.7% (*P* < 0.001) for 1–2 positive LNs and > 2 positive LNs, respectively. The number of positive LNs had prognostic value in cervical squamous cell carcinoma or adenosquamous carcinoma, but not in cervical adenocarcinoma. The number of positive LNs is an independent risk factor for CSS and OS in cervical cancer. This new category might be helpful in better prognostic discrimination of node-positive early stage cervical cancer after hysterectomy.

## INTRODUCTION

Uterine cervical cancer is common worldwide [1]. According to the National Cancer Institute, there were approximately 12,990 new cases diagnosed and 4,120 deaths due to cervical cancer in 2016 [2]. The survival rates for patients with early stage cervical cancer treated with radical hysterectomy in combination with pelvic and/or para-aortic lymphadenectomy were comparable to patients who receive concurrent chemoradiotherapy (CCRT) [3, 4]. However, hysterectomy combined with lymphadenectomy enables clinicians to accurately assess tumor status and guide postoperative adjuvant therapy. Moreover, hysterectomy does not confer the side-effects associated with radiotherapy (RT), such as gastrointestinal, bone and sexual function complications [5].

Lymphadenectomy allows the lymph node status to be accurately assessed. Patients with different numbers of positive lymph nodes (LNs) may represent heterogeneous groups with varied prognoses and clinical outcomes. Previous studies have demonstrated that lymph node status was the main factor that influences survival outcomes in cervical cancer [6–9]. The number of positive LNs is assessed during lymph node staging in a variety of malignant tumors, including breast cancer, esophageal cancer, colorectal cancer, and gastric cancer [10]. However, the American Joint Committee on Cancer (AJCC)/Union for International Cancer Control (UICC) tumor-node-metastasis (TNM) staging system for cervical cancer only considers whether patients have negative or positive lymph node [10]. In addition, lymph node status is not included in the International Federation of Gynecology and Obstetrics (FIGO) staging system [11].

We hypothesize that the number of positive LNs could influence the survival outcomes of patients with early-stage cervical cancer after radical surgery. Therefore, the purpose of this study was to determine the prognostic value of the number of positive LNs in early-stage cervical cancer and to further stratify patients into multiple risk groups using the Surveillance Epidemiology and End Results (SEER) program.

## RESULTS

### Clinicopathological characteristics of the patients

We included 2,222 patients with node-positive early stage cervical cancer. The clinicopathological features of these patients are summarized in Table 1. Median age was 43 (range, 18-88) years. Of the 2,215 patients for whom data on race was available, 80.0% (1773/2215) were Caucasian. In total, 1491 (67.1%), 410 (18.5%), and 198 (8.9%) of the patients had squamous cell carcinoma (SCC), adenocarcinoma (AC), and adenosquamous carcinoma (ASC), respectively. Of the 2,222 patients, 1601 (72.1%) had T1 disease and 621 (27.9%) had T2 cervical cancer. Overall, 1845/2,222 (83.0%) patients received postoperative RT. The median number of removed LNs and positive LNs was 22 (range, 10-88) and 2 (range, 1–32), respectively.

**Table 1:** Clinicopathological characteristics of the 2,222 patients with early-stage cervical cancer

Variable	*N*
Age (years)	
< 50	1518
≥ 50	704
Race (*n* = 2215)	
White	1773
Black	190
Other	252
Tumor histology	
Squamous	1491
Adenocarcinoma	410
Adenosquamous	198
Other	123
Grade (*n* = 2044)	
Well differentiated	95
Moderately differentiated	825
Poorly/undifferentiated	1124
Stage distribution	
T1	1601
IA (FIGO stage)	4
IA1 (FIGO stage)	37
IA2 (FIGO stage)	38
IB (FIGO stage)	686
IB1 (FIGO stage)	489
IB2 (FIGO stage)	172
I NOS (FIGO stage)	175
T2	621
II (FIGO stage)	3
IIA (FIGO stage)	203
IIB (FIGO stage)	415
Number of positive LNs (*n*)	
1–2	1522
> 2	700
Postoperative RT	
No	377
Yes	1845

FIGO, International Federation of Gynecology and Obstetrics; LNs, lymph nodes;

T, tumor; RT, radiotherapy.

### Identification of optimal cut-off points for the number of positive LNs

The optimal cut-off points for the number of positive LNs were identified using receiver operating characteristic (ROC) curve analysis. The analysis showed 2.5 positive LNs was the optimal cut-off point with respect to both cause-specific survival (CSS) (area under the ROC curve [AUC] = 0.594, *P* < 0.001) and and overall survival (OS) (AUC = 0.588, *P* < 0.001). Therefore, we used 1–2 positive LNs and > 2 positive LNs to stratify the patients in order to analyze the association between the number of positive LNs and CSS and OS.

### Prognostic factors

The median follow-up time was 67 months (range, 1–298 months). Five-year and 10-year CSS were 74.7% and 69.3%, respectively; and 5-year and 10-year OS were 72.3% and 65.4%, respectively.

Univariate Cox survival analysis indicated age, tumor stage, tumor histology and the number of positive LNs were significant prognostic factors for both CSS and OS (Table 2). Postoperative RT improved OS (*P* = 0.020) and provided a marginally survival benefit in terms of CSS (*P* = 0.081).

**Table 2:** Univariate analyses of cause specific survival and overall survival

Variable	CSS			OS		
	HR	95% CI	*P*	HR	95% CI	*P*
Age (years)						
< 50	1			1		
≥ 50	1.494	1.264–1.767	< 0.001	1.721	1.476–2.005	< 0.001
Race						
White	1			1		
Black	1.077	0.818–1.418	0.596	1.080	0.837–1.395	0.553
Other	0.954	0.734–1.242	0.728	1.025	0.808–1.299	0.841
Tumor histology						
Squamous	1			1		
Adenocarcinoma	1.917	1.576–2.333	< 0.001	1.728	1.437–2.077	< 0.001
Adenosquamous	1.627	1.248–2.121	< 0.001	1.394	1.081–1.798	0.010
Other	2.889	2.183–3.824	< 0.001	2.466	1.878–3.237	< 0.001
Grade						
Well differentiated	1			1		
Moderately differentiated	0.938	0.608–1.447	0.772	0.895	0.608–1.319	0.576
Poorly/undifferentiated	1.285	0.842–1.961	0.245	1.157	0.793–1.689	0.449
Stage distribution						
T1	1			1		
T2	1.874	1.587–2.213	< 0.001	1.792	1.534–2.092	< 0.001
Number of positive LNs (*n*)						
1–2	1			1		
> 2	1.778	1.510–2.094	< 0.001	1.688	1.450–1.965	< 0.001
Postoperative RT						
No	1			1		
Yes	0.912	0.823–1.011	0.081	0.894	0.814–0.983	0.020

**Figure 1:**
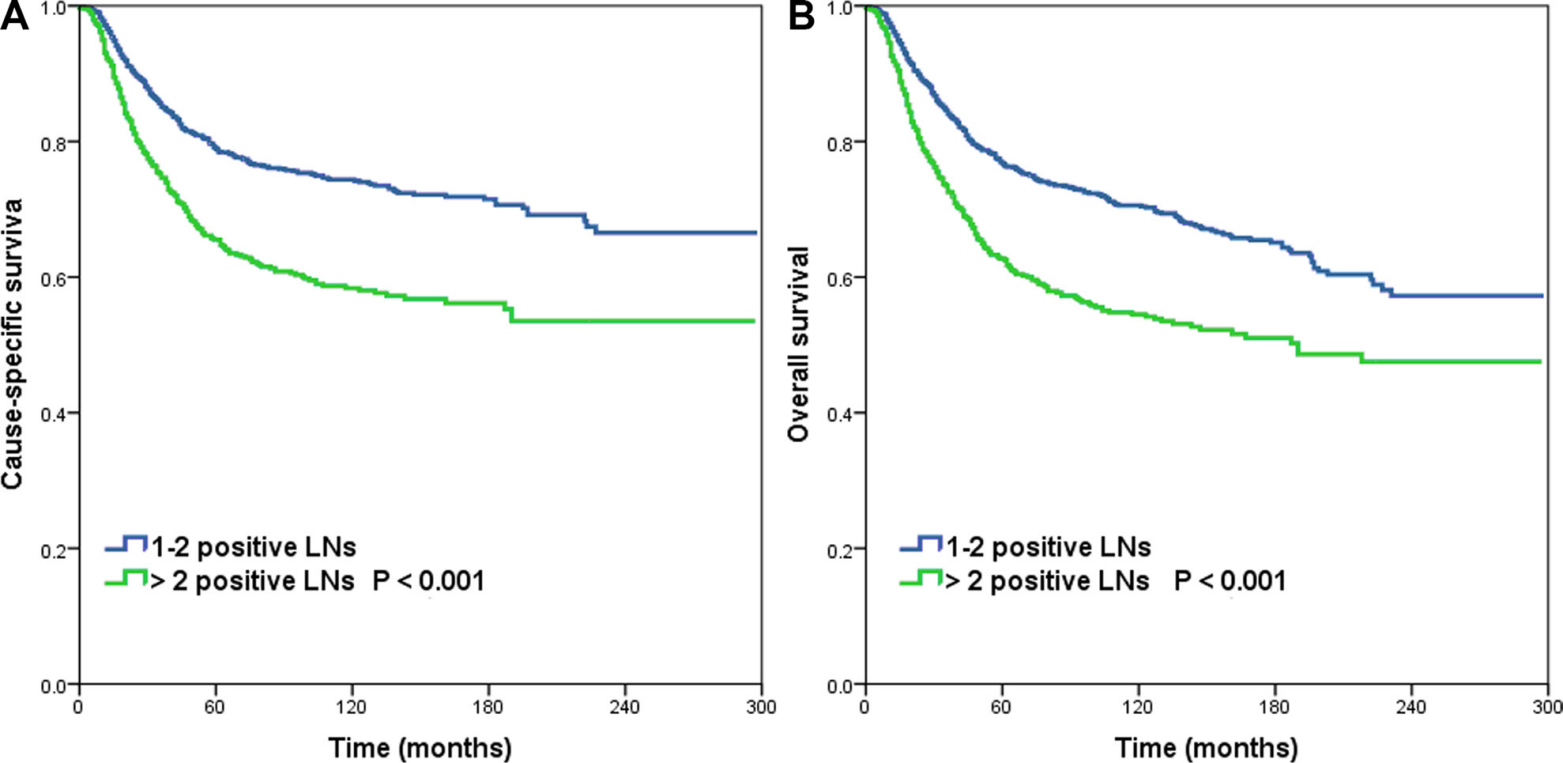
Cause-specific survival (**A**) and overall survival (**B**) for patients with cervical cancer stratified by the number of positive lymph nodes.

Multivariable Cox analysis was used to identify independent prognostic risk factors of survival (Table 3). Patients with > 2 positive LNs had poorer CSS (hazard ratio [HR] 1.631, 95% confidence interval [CI] 1.382–1.926, *P* < 0.001) and OS (HR 1.570, 95% CI 1.346–1.832, *P* < 0.001) compared to patients with 1-2 positive LNs. The 5-year CSS and OS were 78.9% vs. 65.5% (log-rank test, *P* < 0.001) and 76.7% vs. 62.7% (log-rank test, *P* < 0.001) in patients with 1–2 positive LNs and > 2 positive LNs, respectively (Figure 1A–1B). The other prognostic factors that influenced CSS and OS were age, tumor histology, tumor stage and postoperative radiotherapy.

**Table 3:** Multivariate analyses of cause specific survival and overall survival

Variable	CSS			OS		
	HR	95% CI	*P*	HR	95% CI	*P*
Age (years)						
< 50	1			1		
≥ 50	1.334	1.125–1.582	0.001	1.563	1.337–1.826	< 0.001
Tumor histology						
Squamous	1			1		
Adenocarcinoma	1.881	1.545–2.290	< 0.001	1.683	1.399–2.024	< 0.001
Adenosquamous	1.647	1.264–2.147	< 0.001	1.423	1.103–1.834	0.007
Other	2.677	2.021–3.547	< 0.001	2.300	1.751–3.022	< 0.001
Stage distribution						
T1	1			1		
T2	1.660	1.399–1.970	< 0.001	1.564	1.333–1.835	< 0.001
Number of positive LNs (*n*)						
1–2	1			1		
> 2	1.631	1.382–1.926	< 0.001	1.570	1.346–1.832	< 0.001
Postoperative RT						
No	1			1		
Yes	0.883	0.796–1.926	0.019	0.866	0.788–0.953	0.003

CI, confidence interval; CSS, cause-specific survival; LNs, lymph nodes; HR, hazard ratio; OS, overall survival; RT, radiotherapy; T, tumor.

### Impact of tumor stage, tumor histology and the number of positive LNs on survival outcomes

The effect of the number of positive LNs on OS and CSS was analyzed when the patients were stratified by tumor stage and tumor histology. The prognostic value of the number of positive LNs was not affected by tumor stage (both *P* < 0.001). In patients with SCC (CSS, *P* < 0.001; OS, *P* = 0.001) or ASC (CSS, *P* = 0.017; OS, *P* = 0.045) subtypes, the number of positive LNs was also the prognostic factor of survival. However, the number of positive LNs did not have a significant effect on survival among patients with cervical AC (CSS, *P* = 0.215; OS, *P* = 0.184). After 2000, concurrent chemoradiotherapy (CCRT) was rapid adoption for the standard treatment in high-risk early-stage cervical cancer after radical surgery was based on two randomized trials [12, 13]. Our results showed that the number of positive LNs was also the prognostic factor of SCC and ASC subtypes in the era of CCRT, but not in the AC subtype (Table 4).

**Table 4:** Effect of the number of positive lymph nodes on survival according to tumor histology and treatment in the era of concurrent chemoradiotherapy (after 2000)

Variable	CSS			OS		
	HR	95% CI	*P*	HR	95% CI	*P*
Entire group	1.849	1.484–2.305	< 0.001	1.894	1.541–2.327	< 0.001
SCC	2.189	1.609–2.978	< 0.001	2.253	1.708–2.972	< 0.001
AC	1.068	0.690–1.652	0.768	1.115	0.733–1.696	0.612
ASC	2.146	1.090–4.224	0.027	2.041	1.044–3.987	0.037
Without RT	3.191	1.884–5.406	< 0.001	2.880	1.781–4.657	< 0.001
With RT	1.681	1.318–2.413	< 0.001	1.765	1.405–2.217	< 0.001

AC, adenocarcinoma; ASC, adenosquamous carcinoma; CI, confidence interval; CSS, cause-specific survival; HR, hazard ratio; OS, overall survival; RT, radiotherapy; SCC, squamous cell carcinoma.

Next, the patients were classified as four subgroups according to tumor stage and the number of positive LNs. T1 and 1–2 positive LNs, T1 and > 2 positive LNs, T2 and 1–2 positive LNs, and T2 and >2 positive LNs were defined as T1N1, T1N2, T2N1, and T2N2, respectively. In the entire cohort of 2,222 patients, the T1N1 group achieved the best CSS and OS rates, the T2N2 group had the poorest survival rates, and patients in the T2N1 and T1N2 groups had similar CSS and OS rates (CSS, *P* = 0.771; OS, *P* = 0.704). The survival of these four groups were similar to entire group in SCC disease. However, among patients with ASC subtype, the survival curves of the T1N1, T1N2 and T2N1 groups overlapped (CSS, *P* = 0.973; OS, *P* = 0.913) while the T2N2 group had significantly poorer survival than the other three groups.

**Figure 2:**
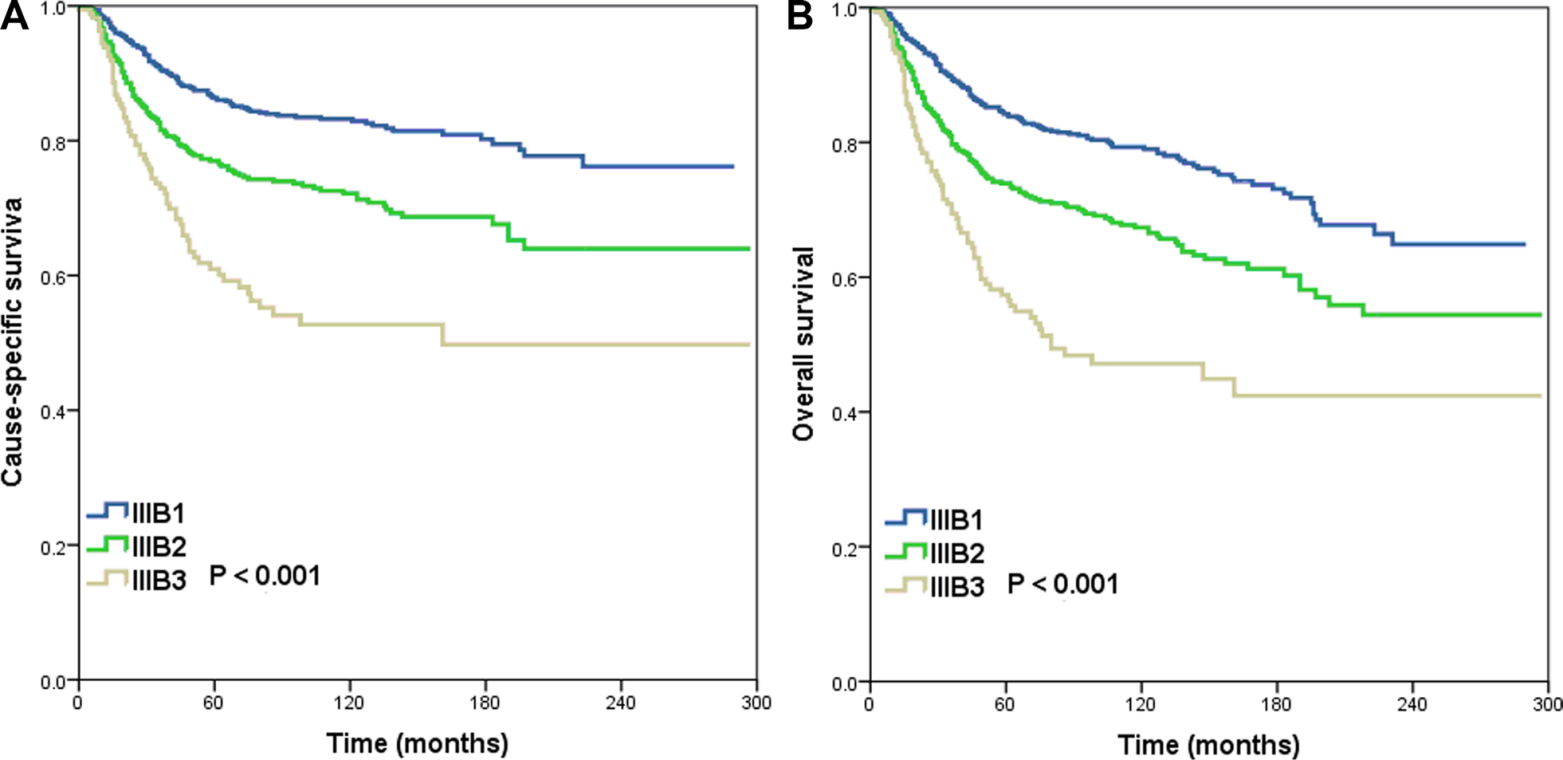
Cause-specific survival (**A**) and overall survival (**B**) for patients with cervical squamous cell carcinoma using the proposed revised TNM classification system.

**Figure 3:**
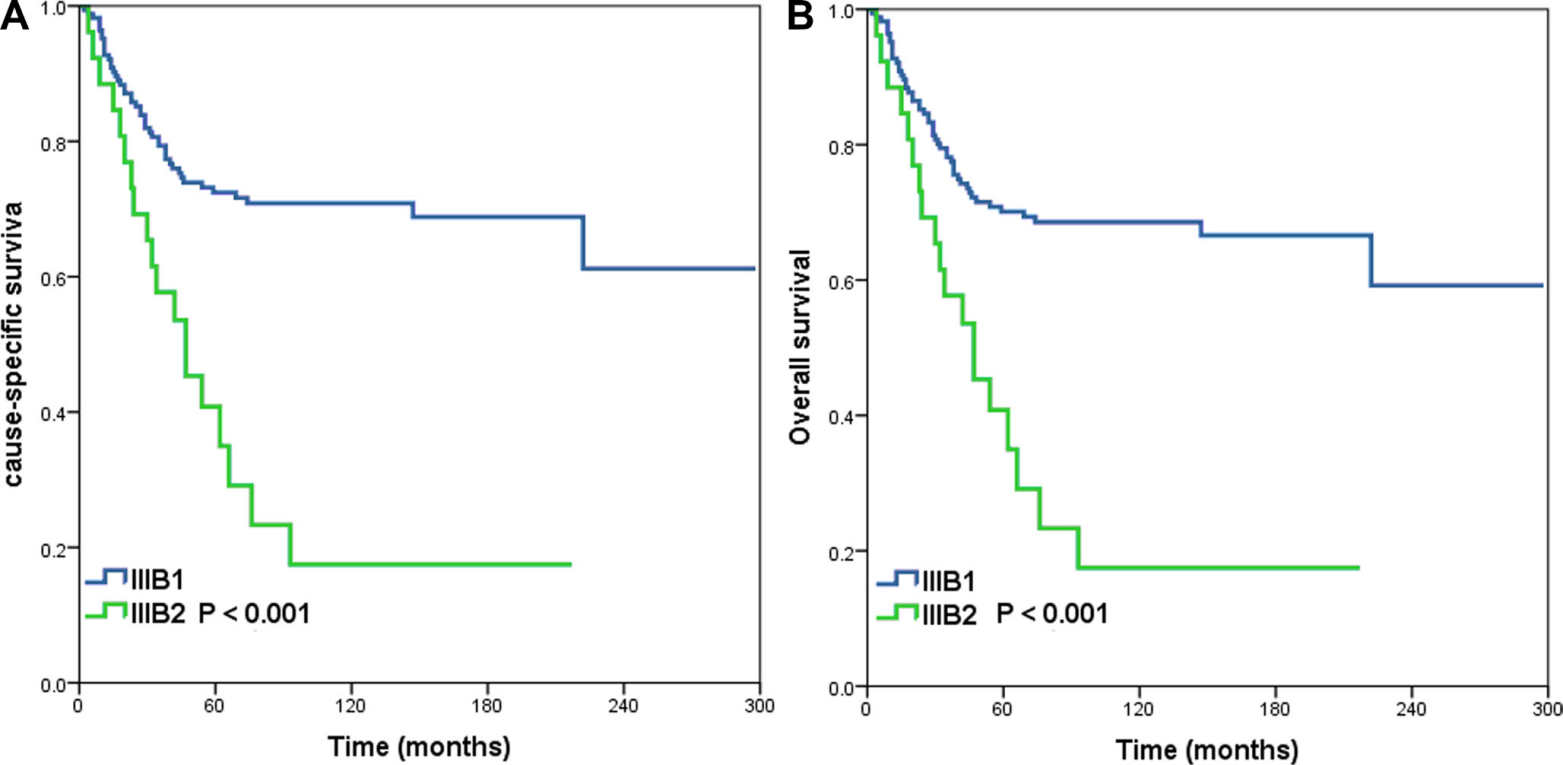
Cause-specific survival (**A**) and overall survival (**B**) for patients with cervical adenosquamous carcinoma using the proposed revised TNM classification system.

Based on the results of these analyses, we recommend a revised TNM staging system that accounts for tumor stage and the number of positive LNs (Table 5). In SCC patients, T1N1 should be defined as stage IIIB1, T1N2 and T2N1 as stage IIIB2, and T2N2 as stage IIIB3 (Figure 2A–2B). For patients with ASC, stage T1N1, T1N2 and T2N1 should be defined as stage IIIB1, and T2N2 as stage IIIB2 (Figure 3A–3B).

**Table 5:** Proposed revised TNM classification system for cervical cancer incorporating the number of positive lymph nodes and tumor histology

Current TNM stage		Entire cohort		Squamous		Adenosquamous	
IIIB	T-3N1	IIIB1	T1N1	IIIB1	T1N1	IIIB1	T1N1
		IIIB2	T1N2	IIIB2	T1N2		T1N2
			T2N1		T2N1		T2N1
		IIIB3	T2N2	IIIB3	T2N2	IIIB2	T2N2

### Impact of postoperative RT and the number of positive LNs on survival outcomes

The clinical value of the number of positive LNs was further analyzed in patients who were treated with and without postoperative radiotherapy. There were no differences between the non-RT group and RT group in race, age, tumor stage, grade, tumor histology, lymph node metastasis as a categorical variable or as a continuous variable. The prognostic value of the number of positive LNs was not affected by postoperative RT. Patients with > 2 positive LNs had poorer CSS and OS in both the non-RT group (CSS, *P* < 0.001; OS, *P* < 0.001) and RT group (CSS, *P* < 0.001; OS, *P* < 0.001) compared to the respective groups of patients with 1–2 positive LNs, and the results were similar in the era of CCRT (Table 4).

## DISCUSSION

In this study, we used the SEER database to evaluate the prognostic value of the number of positive LNs in node-positive early stage cervical cancer after hysterectomy. Our results showed that a higher number of positive LNs was associated with adverse survival outcomes.

The clinical value of the number of positive LNs in node-positive early stage cervical cancer remains controversial. In a study of 141 patients with FIGO IB-IIB cervical cancer, Kasuya et al. reported that patients with 1–2 positive LNs had better survival outcomes than patients with > 2 positive LNs [14]. Similarly, Horn et al. also found that patients with < 3 positive LNs had better OS than patients with > 3 positive LNs [15]. However, Park et al. did not found association between the number of positive LNs and the survival outcomes in an analysis of 188 patients with FIGO IA-IIA cervical cancer [16]. In order to reduce the potential impact of variation in the number of lymph nodes dissected, in this study we only assessed patients who had more than 10 lymph nodes removed. We found that patients with > 2 positive LNs had significantly poorer CSS and OS than patients with 1–2 positive LNs. Therefore, it is necessary to determine the number of positive LNs in order to accurately assess the prognosis of patients with cervical cancer.

The number of positive LNs is one of the important factors in the TNM staging system in a variety of malignancies. However, even in the present edition, the TNM staging system for cervical cancer only considers whether patients are lymph node negative or positive. Moreover, the impact of lymph node status has not been considered with respect to the FIGO staging system [11]. Based on the current TNM staging system, T1-3N1 disease is defined as FIGO stage IIIB cervical cancer [10]. The data presented in this study indicates that the number of positive LNs should be incorporated into the TNM staging system of cervical cancer.

Postoperative radiotherapy is a standard adjuvant treatment for node-positive cervical cancer [17]. In this study, 83% of patients received postoperative RT, and multivariable analysis suggested postoperative RT improved CSS and OS. In addition, the prognostic value of the number of positive LNs was not affected by postoperative RT. In a study by Hosaka et al. also found that patients with > 2 positive LNs experienced poorer survival in 108 patients with FIGO IB-IIIB cervical cancer [18]. However, it is unknown whether postoperative CCRT could influence the prognostic value of the number of positive LNs. Okazawa et al. studied 119 patients with FIGO IB1-IIB cervical cancer and found that the number of positive LNs (> 2 vs. 1–2 positive LNs) only had clinical value in patients who received postoperative RT (*n* = 67), but not in adjuvant CCRT (*n* = 70) [19]. We were unable to extract information from the study by Okazawa et al. on how treatment decisions were made, such as the combination of postoperative RT with CCRT [19]. Previous studies indicated that adjuvant CCRT with cisplatin is superior to RT alone in terms of both OS and progression-free survival in node-positive cervical cancer [13, 20]. As the data in this study was obtained using the SEER database, we were unable to obtain precise information on chemotherapy regimens for this cohort. As the CCRT was rapid adoption for standard treatment for high-risk early-stage cervical cancer who undergo radical surgery after the year 2000 [12, 13]. Our further analysis found that in the era of CCRT, the number of positive LNs was also a prognostic factor for survival. Therefore, it will be important to further investigate the effectiveness of adjuvant therapy for patients with multiple positive LNs.

Cervical SCC, ASC and AC exhibit different biological behaviors and varied clinical outcomes [21, 22]. Research into the number of positive LNs in different histological types of cervical cancer is limited. Liu et al. reported that > 2 positive LNs and multiple groups of pelvic positive LNs appeared to identify patients with poorer survival outcomes in node positive early stage SCC of the cervix (*n* = 296) [23]. The results of Hosaka et al. showed that the OS was 86.8%, 25.0%, and 0 for patients with 1 or 2 positive LNs irrespective of histologic subtype (*n* = 68), > 2 positive LNs in SCC and ASC subtypes (*n* = 32), and > 2 positive LNs in AC disease (*n* = 8), respectively [18]. In the study of cervical AC by Kato et al., patients with 1 positive LN (*n* = 7) achieved better 5-year OS than patients with > 2 positive LNs (*n* = 27) (86% vs. 23%, *P* = 0.009) [24]. However, it is worth noting that the small numbers of patients with AC in the studies by the above two studies. In our study, we observed that the number of positive LNs only had prognostic value in SCC and ASC subtypes, but not in AC subtype. We were unable to identify reasons why the number of positive LNs lacked clinical value in AC, though this may be associated with the relative biological aggressiveness and resistance to adjuvant therapy of AC compared to SCC [25].

We must acknowledge several limitations of our study. First, registry-based retrospective study may be inherently biased toward heterogeneous patient population. Secondly, the SEER does not include a variety of information, including the chemotherapy regimen and dose; the use of preoperative, concurrent and adjuvant chemotherapy; margin status, and local or distant recurrence. In addition, the patients assessed in this study were treated during a period of 24 years, during which therapeutic regimens and pathological evaluation methods are likely to have changed. However, the strength of this study is its analysis of data on a large number of patients from the SEER program, which was specifically set-up to provide population-based data.

In conclusion, we demonstrate that the number of positive LNs is an independent risk factor for survival outcomes in node-positive early stage cervical cancer. This new category might be helpful in better prognostic discrimination of patients. Further prospective trials are warranted to confirm the value of stratifying patients based on the number of positive LNs.

## MATERIALS AND METHODS

### Patients

Patients diagnosed with cervical cancer between 1988 and 2012 were included using the SEER database [26]. Patients were included in this study based upon the following criteria: 1) FIGO stage IA-IIB uterine cervical cancer, 2) patients who received hysterectomy and lymphadenectomy with more than 10 lymph nodes removed, 3) and patients with pathologically-confirmed positive LNs. Patients whose lymph nodes were not examined or patients with an unknown number of positive LNs were excluded. This study was approved by the ethics committee of the First Affiliated Hospital of Xiamen University and Sun Yat-sen University Cancer Center.

### Clinicopathological factors

The following clinicopathological variables were collected for analysis: age, race, tumor stage, FIGO stage, grade, histology, number of positive LNs, number of removed LNs, and postoperative RT. Tumor stage was defined according to the UICC/AJCC staging system (8). The T1 stage included stages IA, IA1, IA2, IB, IB1, IB3, and I not otherwise specified (NOS) of the FIGO staging system. Patients with FIGO stage II, IIA, and IIB cervical cancer were classified as T2 disease. The primary outcomes of this study were CSS and OS.

### Statistical analysis

Categorical variables were analyzed using the Pearson's χ2 (chi-squared) test and continuous variables with *T*-tests and one-way ANOVA. The optimum cut-off point for the number of positive LNs was determined using ROC curves. Survival rates were plotted using the Kaplan-Meier method and compared using the log-rank test. Survival analyses were performed using univariate and multivariate Cox regression analyses. Risk factors that were considered to be of potential importance in univariate analysis were included in the multivariate analysis. All statistical analyses were performed with statistical software package SPSS (version 21.0; IBM Corporation, Armonk, NY, USA). *P* < 0.05 was considered statistically significant in all analyses.

## References

[1] Waggoner SE (2003). Cervical cancer. Lancet.

[2] Siegel RL, Miller KD, Jemal A (2016). Cancer statistics, 2016. CA Cancer J Clin.

[3] Perez CA, Camel HM, Kao MS, Hederman MA (1987). Randomized study of preoperative radiation and surgery or irradiation alone in the treatment of stage IB and IIA carcinoma of the uterine cervix: final report. Gynecol Oncol.

[4] Landoni F, Maneo A, Colombo A, Placa F, Milani R, Perego P, Favini G, Ferri L, Mangioni C (1997). Randomised study of radical surgery versus radiotherapy for stage Ib-IIa cervical cancer. Lancet.

[5] Viswanathan AN, Lee LJ, Eswara JR, Horowitz NS, Konstantinopoulos PA, Mirabeau-Beale KL, Rose BS, von Keudell AG, Wo JY (2014). Complications of pelvic radiation in patients treated for gynecologic malignancies. Cancer.

[6] Trattner M, Graf AH, Lax S, Forstner R, Dandachi N, Haas J, Pickel H, Reich O, Staudach A, Winter R (2001). Prognostic factors in surgically treated stage ib-iib cervical carcinomas with special emphasis on the importance of tumor volume. Gynecol Oncol.

[7] Suprasert P, Srisomboon J, Kasamatsu T (2005). Radical hysterectomy for stage IIB cervical cancer: a review. Int J Gynecol Cancer.

[8] Zhao H, Li L, Su H, Lin B, Zhang X, Xue S, Fei Z, Zhao L, Pan Q, Jin X, Xie C (2016). Concurrent paclitaxel/cisplatin chemoradiotherapy with or without consolidation chemotherapy in high-risk early-stage cervical cancer patients following radical hysterectomy: preliminary results of a phase III randomized study. Oncotarget.

[9] Zheng RR, Huang M, Jin C, Wang HC, Yu JT, Zeng LC, Zheng FY, Lin F (2016). Cervical cancer systemic inflammation score: a novel predictor of prognosis. Oncotarget.

[10] International Union (2011). Against Cancer TNM classification of malignant tumours.

[11] Odicino F, Pecorelli S, Zigliani L, Creasman WT (2008). History of the FIGO cancer staging system. Int J Gynaecol Obstet.

[12] Keys HM, Bundy BN, Stehman FB, Muderspach LI, Chafe WE, Suggs CL, Walker JL, Gersell D (1999). Cisplatin, radiation, and adjuvant hysterectomy compared with radiation and adjuvant hysterectomy for bulky stage IB cervical carcinoma. N Engl J Med.

[13] Peters WA, Liu PY, Barrett RJ, Stock RJ, Monk BJ, Berek JS, Souhami L, Grigsby P, Gordon W, Alberts DS (2000). Concurrent chemotherapy and pelvic radiation therapy compared with pelvic radiation therapy alone as adjuvant therapy after radical surgery in high-risk early-stage cancer of the cervix. J Clin Oncol.

[14] Kasuya G, Ogawa K, Iraha S, Nagai Y, Hirakawa M, Toita T, Kakinohana Y, Kudaka W, Inamine M, Ariga T, Aoki Y, Murayama S (2013). Postoperative radiotherapy for uterine cervical cancer: impact of lymph node and histological type on survival. Anticancer Res.

[15] Horn LC, Hentschel B, Galle D, Bilek K (2008). Extracapsular extension of pelvic lymph node metastases is of prognostic value in carcinoma of the cervix uteri. Gynecol Oncol.

[16] Park JY, Kim DY, Kim JH, Kim YM, Kim YT, Nam JH (2010). Further stratification of risk groups in patients with lymph node metastasis after radical hysterectomy for early-stage cervical cancer. Gynecol Oncol.

[17] Wolfson AH, Varia MA, Moore D, Rao GG, Gaffney DK, Erickson-Wittmann BA, Jhingran A, Mayr NA, Puthawala AA, Small W, Yashar CM, Yuh W, Cardenes HR, American College of Radiology (ACR) (2012). ACR Appropriateness Criteria® role of adjuvant therapy in the management of early stage cervical cancer. Gynecol Oncol.

[18] Hosaka M, Watari H, Mitamura T, Konno Y, Odagiri T, Kato T, Takeda M, Sakuragi N (2011). Survival and prognosticators of node-positive cervical cancer patients treated with radical hysterectomy and systematic lymphadenectomy. Int J Clin Oncol.

[19] Okazawa M, Mabuchi S, Isohashi F, Suzuki O, Ohta Y, Fujita M, Yoshino K, Enomoto T, Kamiura S, Kimura T (2012). The prognostic significance of multiple pelvic node metastases in cervical cancer patients treated with radical hysterectomy plus adjuvant chemoradiotherapy. Int J Gynecol Cancer.

[20] Ryu HS, Chun M, Chang KH, Chang HJ, Lee JP (2005). Postoperative adjuvant concurrent chemoradiotherapy improves survival rates for high-risk, early stage cervical cancer patients. Gynecol Oncol.

[21] Noh JM, Park W, Kim YS, Kim JY, Kim HJ, Kim J, Kim JH, Yoon MS, Choi JH, Yoon WS, Kim JY, Huh SJ (2014). Comparison of clinical outcomes of adenocarcinoma and adenosquamous carcinoma in uterine cervical cancer patients receiving surgical resection followed by radiotherapy: a multicenter retrospective study (KROG 13–10). Gynecol Oncol.

[22] Zhou J, Wu SG, Sun JY, Li FY, Lin HX, Chen QH, He ZY (2017). Comparison of clinical outcomes of squamous cell carcinoma, adenocarcinoma, and adenosquamous carcinoma of the uterine cervix after definitive radiotherapy: a population-based analysis. J Cancer Res Clin Oncol.

[23] Liu Y, Zhao LJ, Li MZ, Li MX, Wang JL, Wei LH (2015). The Number of Positive Pelvic Lymph Nodes and Multiple Groups of Pelvic Lymph Node Metastasis Influence Prognosis in Stage IA-IIB Cervical Squamous Cell Carcinoma. Chin Med J (Engl).

[24] Kato T, Watari H, Takeda M, Hosaka M, Mitamura T, Kobayashi N, Sudo S, Kaneuchi M, Kudo M, Sakuragi N (2013). Multivariate prognostic analysis of adenocarcinoma of the uterine cervix treated with radical hysterectomy and systematic lymphadenectomy. J Gynecol Oncol.

[25] Kodama J, Seki N, Ojima Y, Nakamura K, Hongo A, Hiramatsu Y (2006). Prognostic factors in node-positive patients with stage IB-IIB cervical cancer treated by radical hysterectomy and pelvic lymphadenectomy. Int J Gynaecol Obstet.

[26] Surveillance, Epidemiology, and End Results (SEER) Program (www.seer.cancer.gov) SEER*Stat Database: Incidence-SEER 9 Regs Research Data, Nov 2015 Sub (1973–2013) <Katrina/Rita Population Adjustment> -Linked To County Attributes-Total U.S., 1969–2014 Counties, National Cancer Institute, DCCPS, Surveillance Research Program, Surveillance Systems Branch, released April 2016, based on the November 2015 submission. http://www.seer.cancer.gov.

